# Factors Influencing Rural End-Users' Acceptance of e-Health in Developing Countries: A Study on Portable Health Clinic in Bangladesh

**DOI:** 10.1089/tmj.2018.0039

**Published:** 2019-03-18

**Authors:** Nazmul Hossain, Fumihiko Yokota, Nazneen Sultana, Ashir Ahmed

**Affiliations:** ^1^ Department of Advanced Information Technology, Kyushu University, Fukuoka, Japan.; ^2^ Department of Marketing, University of Dhaka, Dhaka, Bangladesh.; ^3^ Institute of Decision Science for Sustainable Society (IDS3), Kyushu University, Fukuoka, Japan.; ^4^ Grameen Communications, Dhaka, Bangladesh.

**Keywords:** *e-health*, *end-users' acceptance*, *TAM*, *developing countries*

## Abstract

**Background:** Existing studies regarding e-health are mostly focused on information technology design and implementation, system architecture and infrastructure, and its importance in public health with ancillaries and barriers to mass adoption. However, not enough studies have been conducted to assess the end-users' reaction and acceptance behavior toward e-health, especially from the perspective of rural communities in developing countries.

**Objective:** The objective of this study is to explore the factors that influence rural end users' acceptance of e-health in Bangladesh.

**Methods:** Data were collected between June and July 2016 through a field survey with structured questionnaire form 292 randomly selected rural respondents from Bheramara subdistrict, Bangladesh. Technology Acceptance Model was adopted as the research framework. Logistic regression analysis was performed to test the theoretical model.

***Results:** The study found social reference as the most significantly influential variable (Coef. = 2.28, odds ratio [OR] = 9.73,* p *< 0.01) followed by advertisement (Coef. = 1.94, OR = 6.94,* p *< 0.01); attitude toward the system (Coef. = 1.52, OR = 4.56,* p *< 0.01); access to cellphone (Coef. = 1.37, OR = 3.92,* p *< 0.05), and perceived system effectiveness (Coef. = 0.74, OR = 2.10,* p *< 0.01). Among demographic variables, age, gender, and education were found significant while we did not find any significant impact of respondents' monthly family expenditure on their e-health acceptance behavior. The model explains 54.70% deviance (R^2^ = 0.5470) in the response variable with its constructs. The “Hosmer-Lemeshow” goodness-of-fit score (0.539) is also above the standard threshold (0.05), which indicates that the data fit well with the model.*

**Conclusion:** The study provides guidelines for the successful adoption of e-health among rural communities in developing countries. This also creates an opportunity for e-health technology developers and service providers to have a better understanding of their end users.

## Introduction

Remote healthcare systems, including e-health, m-health, telemedicine, telemonitoring, electronic health records, and hospital information systems, are getting attention due to the rapid advancement in information and communication technology (ICT) worldwide.^1^ However, some rural and remote communities especially in developing and underdeveloped countries are still deprived of quality healthcare services due to the lack of necessary infrastructure, insufficiently qualified healthcare workforce, and expensive access to quality healthcare.^2,3^ In this circumstance, the concept of e-health has emerged and gained momentum. Dansky et al.^4^ described e-health as one of the most prominent contributions of ICT toward healthcare with noticeable positive impacts. DeLuca and Enmark^5^ defined e-health as an umbrella that includes a spectrum of technologies, including computers, telephony, and wireless communications to provide healthcare access to remote patients, care providers, care management, and educators. Oh et al.^6^ defined e-health as a process of providing medical assistance through electronic means, in particular through the Internet, which includes teaching, treating, monitoring, and interacting with patients as well as health professionals.

### Portable Health Clinic

Portable Health Clinic (PHC) is an e-health initiative, jointly developed by Kyushu University, Japan and Grameen Communications, Bangladesh to provide affordable healthcare solutions to low-income, low-literate people living in remote and underserved communities in Bangladesh by using ICT.^7,8^

To get healthcare services from PHC, a patient first has to register his/her vital information such as name, age, gender, location, and disease complaints with the PHC system, which generates a unique patient ID. Second, a health checkup is conducted with an assistance of healthcare worker; checkup data are automatically sent and stored in a central PHC server. The third step is that a teleconsultancy (voice and video) between the patient in need and the remote doctor is located at the PHC. After having the conversation with patients and analyzing their clinical data, if necessary, the doctor might issue an e-prescription, and a printed version of that e-prescription is given to the patient.

The PHC began its experimental service in 2010. Through January 31, 2018, the service has reached 32 remote locations in 9 districts and served 41,240 rural patients of which 55.2% were male and 44.8% female.^9^ For our research, we selected Bheramara subdistrict of Kushtia as our data collection site, which is one of the above mentioned nine districts, located in the northwestern part of Bangladesh. PHC started its service in Bheramara in 2012 and served 4,701 rural patients through January 2018.

## Research Motivation and Objective

Research related to e-health and health information technology (IT) often focuses more on IT design and implementation,^10^ and probably not enough on how the end users react toward already implemented IT.^11^ The success of health IT does not only depend on its design and infrastructure but also on its end-users' acceptance for whom the service is being designed.^12^ However, some recent studies explored that despite assuming potential benefits, the adoption rate of e-health is insignificant in Bangladesh, especially among rural inhabitants. Ahmed et al.^13^ found that people were somehow aware of e-health and considered it as a potentially useful service, however, a very few had actually used it. Khan et al.^14^ found expensive consultation fees, lack of technical knowledge to operate the system, and lack of trust in unknown physicians are the leading obstacles to adopt e-health in Bangladesh.

Hoque and Bao^15^ investigated the influence of cultural dimensions on the adoption of e-health in urban society in Bangladesh. Khatun et al.^16^ found illiteracy, lack of English language proficiency, lack of trust, and technological incapability impeding rural Bangladeshi communities to adoption of e-health and m-health, whereas a sense of ownership, evidence of utility, a positive attitude, and intention of future were driving forces in the adoption process. Hoque and Sorwar^17^ investigated the underlying factors influencing the adoption of m-health by urban elderly in Bangladesh. Hossain et al.^18^ investigated demographic and socioeconomic factors that affect rural inhabitants' acceptance of e-health in Bangladesh.

The aforementioned literature review depicts most of the existing studies that are focused on IT design and implementation, system architecture, and infrastructural issues of e-Health. Some described the importance of e-health in public health development, including its ancillaries and barriers, to mass adoption.^13–16^ Some studies separately measured the impact of cultural factors,^15^ demography, and socioeconomic factors^18^ on e-health adoption. However, not enough studies are conducted to explore the rural end-users' acceptance of e-health, especially from the perspective of developing nations. It is necessary to measure the combined impact of demographic, behavioral, technical, and promotional factors on end-users' acceptance of e-health. The objective of this study is to explore the factors that affect rural end-users' acceptance of e-health in a developing country such as Bangladesh.

## Research Framework and Hypothesis

In last three decades, several theories have been developed to explain the factors affecting individuals' acceptance of new technologies or technology-based services. The most renowned and frequently used theories are Technology Acceptance Model (TAM), TAM 2, Theory of Planned Behavior (TPB), Theory of Reasoned Action (TRA), Combined TAM and TPB, and the Unified Theory of Acceptance and Use of Technology (UTAUT). TAM first was established by Fred Davis in 1989 as a theory of information system that models how users understand, approach, utilize, come to accept, and use a technology.^19^ TAM2 added cognitive and social influences to predict technology acceptance. The cognitive aspect included perceived ease of use (PEU), job relevance, quality of output, and results demonstrability. While social influences focused mainly on subjective norms and voluntariness.^20^ TPB originally evolved from the TRA with an added variable perceived behavior control.^21^ The UTAUT model derived by comprehensive examination of various models mentioned above aiming to achieve a unified view of user acceptance.^22^

**Fig. 1.**
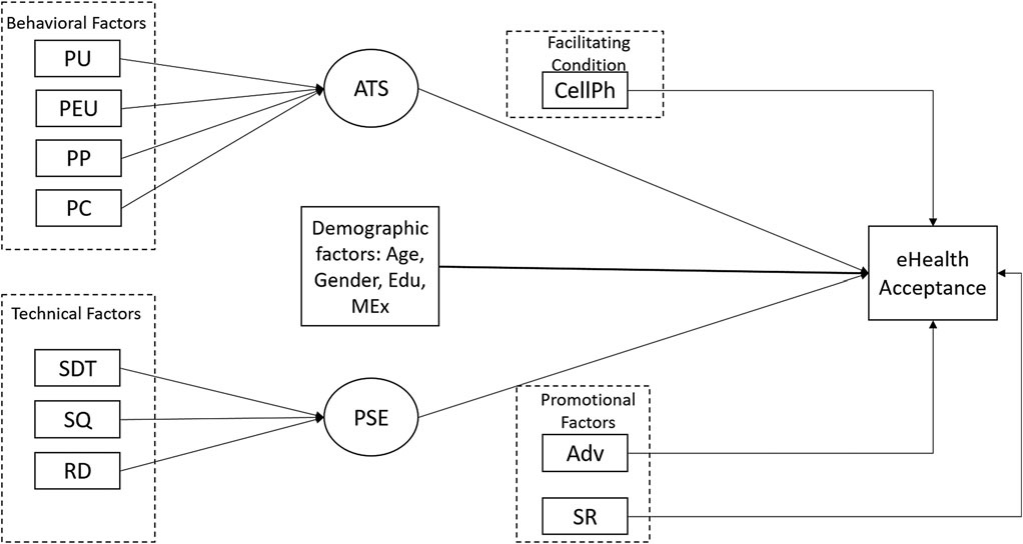
Research framework. Adv, advertisements; ATS, attitude toward the system; CellPh, cellphone ownership; Edu, education; MEx, monthly family expenditure; PC, perceived cost; PEU, perceived ease of use; PP, perceived privacy; PSE, perceived system effectiveness; PU, perceived usefulness; RD, result demonstrability; SDT, service delivery time; SQ, service quality; SR, social reference.

To attain the research objective, this study adopts TAM, which is the most notable model to explain end-users' behavior in health IT.^23^ Although TAM is widely used in healthcare research, it should be kept in mind that this model is not developed solely for healthcare. Researchers have applied this model in e-commerce,^24^ e-banking,^25^ online ticketing system,^26^ office productivity software,^27^ social media,^28^ virtual archive,^29^ and in many other IT product or services. Thus, Holden and Karsh^11^ suggested, if TAM is used in its generic form, it may not capture some of the unique contextual features of IT-based healthcare delivery systems. This is why we proposed an extension of TAM, shown in *Figure 1* as our research framework.

In this proposed framework, perceived usefulness (PU), perceived ease of use, and attitude toward the system (ATS) were adopted from the original TAM proposed by Davis.^19^ Service quality (SQ) and result demonstrability (RD) were adopted from TAM2.^20^ Social reference (SR) and facilitating condition were adopted from UTAUT.^22^ The model also incorporates a few additional variables based on existing literature and empirical evidences to have a better understanding of consumers' acceptance behavior toward e-health, which are perceived cost (PC),^30^ perceived privacy (PP),^31^ service delivery time (SDT),^32^ perceived system effectiveness (PSE),^33^ and impact of advertisement (Adv).^34^

### Demography

Several studies^35–37^ confirmed the profound impact of demographic factors such as age, gender, income, and education on consumers' buying behavior and decision-making process. Thus, we also wanted to explore the impact of demography on rural end users' acceptance of e-health.

**H1.** Age has a positive impact on end-users' acceptance of e-health

**H2.** Gender has an impact on end-users' acceptance of e-health

**H3.** Level of education has a positive impact on end-users' acceptance of e-health

**H4.** Monthly family expenditure has a positive impact on end-users' acceptance of e-health

### Attitude

Davis^19^ defined attitude as individual's evaluative judgment of the target behavior on some dimension (e.g., good/bad, harmful/beneficial, pleasant/unpleasant). Ajzen et al.^38^ defined attitude as the degree to which a person likes or dislikes any object. In our study, we considered attitude as a latent variable, which has been extracted from four other observed variables namely PU, PEU, PP, and PC.

**H5.** Attitude toward e-health has a favorable impact on end-users' acceptance of e-health

### Perceived System Effectiveness

System effectiveness has been defined as the extent to which a system can be expected to achieve its goals within its specific environment. The effectiveness of the system depends on its output quality, visibility, timeliness, and reliability.^33,39^ In our study, we considered PSE as a latent variable which has been extracted from three more observed variables, namely SDT, SQ, and RD.

**H6.** PSE has a positive influence on end-users' acceptance of e-health

### Facilitating Condition

It refers to the factors that facilitate or encourage a particular behavior to occur in a given environment. It also includes an existing technical infrastructure to support using any new system.^22^ In our study, we considered cellphone ownership as a facilitator of using e-health.

**H7.** Cellphone ownership positively influences the acceptance of e-health

### Promotion

Promotion, in marketing, is any type of communication aimed to inform the relative merits of a product or service to its target audiences and encourage them to use it. The goal of promotion is to increase awareness, create interest, and finally generate sales.^40^ Promotion plays a vital role in healthcare initiatives to be accepted by its target consumers.^34^ Since PHC does periodic promotional campaigns in its service area, we are interested to see its impact on consumers' response.

**H8.** Exposed to Adv has a positive impact on end-users' acceptance of e-health

### Social Reference

People become influenced when someone considered to be important to them refers any particular product or service or encourages to exhibit a given behavior.^20,22^ The referee can be anyone from friends and family, coworkers, or acquaintance.

**H9.** SR has a positive impact on end-users' acceptance of e-health

In this study, we consider end-users' acceptance as the response of actual users who received any healthcare service from a PHC system at least once.

## Research Methodology

The study is exploratory^41^ and quantitative in nature. Data were collected between June and July 2016 through a field survey conducted in Bheramara subdistrict of Kushtia, a northwestern district of Bangladesh. A structured questionnaire was developed initially in English, and later translated into Bengali. Close-ended questions were used to extract respondents' demography and a 5-point Likert scale from extremely disagree to extremely agree with a neutral point on 3 was used to extract the cognitive information. A pilot study was conducted with seven randomly selected <18-year-old rural inhabitants to test the understandability of the questionnaire. Their feedback was considered to review the questionnaire. To maintain the right of privacy of the respondents, they were briefed on the research purpose and asked whether they want to participate in the survey as well as allow us to use their response in our scientific publications.

A total of 597 questionnaires were distributed randomly, however, after deducting missing fields and partially answered questionnaires, we could include 292 respondents as our effective sample. The sample was drawn by a simple random sampling method, which eliminates the bias by giving all individuals an equal chance to be chosen.^42^ There is a variety of opinions regarding the optimum sample size for different types of statistical analyses. According to Bartlett et al.^43^ a sample of 200 as fair and 300 as good for statistical analysis, including logistic regression modeling. Malhotra^44^ suggested 200 as a critical sample size that can be used in any common estimation procedure for valid results. Kenny^45^ suggested that, in behavioral science with multivariate analysis, the sample size should be at least 10 times the number of items in the study. In our study, the model consists of 15 items, including both independent and dependent. As per above studies, we considered a sample size of 292 as optimum for our study.

Lee et al.^46^ conducted a meta-analysis on 101 TAM oriented articles published in leading IS journals and conferences from 1986 to June 2003 and found 87% research conducted the nonlongitudinal study, 85% collected data through field survey, and 32% research used regression modeling as their analysis method. Our proposed model is constructed with logistic regression. Data were collected through field survey with a structured questionnaire and analyzed with logistic regression and other statistical tools, including principal component analysis, factor analysis, reliability test, and Pearson correlation test.

## Results

### Respondents' Demography

Respondents' demographic characteristics are shown in *Table 1* (Sample demographics, *n* = 292).

**Table 1.** Sample Demographics^*^

	FREQUENCY	PERCENTAGE
Gender		
Male	205	70.0
Female	87	30.0
Age group		
Less than 30	67	22.9
30–45	148	50.6
46–60	64	21.9
More than 60	13	4.5
Education		
None	23	8.0
Primary	72	25.0
Secondary	114	39.0
College and higher	83	28.0
Monthly family expenditure (in BDT)		
Less than 6,000	31	11.0
6,001–10,000	118	40.0
10,001–15,000	100	34.0
15,001–20,000	30	10.0
More than 20,000	13	4.0
Cellphone ownership		
Yes	252	86.0
No	40	14.0
e-health (PHC) use		
Yes	171	58.0
No	121	42.0

^*^ (*n* = 292).

BDT, Bangladeshi taka (the local currency of Bangladesh); PHC, Portable Health Clinic.

### Descriptive Statistics of Observed Variables

As mentioned earlier, a 5-point Likert scale was used to measure the cognitive aspects of the respondents, that is, their perception and attitude toward e-health. To do so, seven observed variables have been measured and the descriptive statistics are shown in *Table 2*.

**Table 2.** Descriptive Statistics of Observed Variables

	PU	PEU	PP	PC	SDT	SQ	RD
Mean	3.52	3.29	3.18	3.82	3.68	3.32	3.26
Standard Deviation	0.91	0.70	0.66	1.10	0.83	0.75	0.68
Count	292	292	292	292	292	292	292

PC, perceived cost; PEU, perceived ease of use; PP, perceived privacy; PU, perceived usefulness; RD, result demonstrability; SDT, service delivery time; SQ, service quality.

These statistics showed a PC and SDT, which received the most favorable response, while privacy and RD remained less favorable. In other words, respondents considered PHC service cost as cheaper and delivery time as faster than that of other existing traditional healthcare services. On the contrary, people are concerned about their privacy and result understandability while using e-health.

### Extraction of Latent Variables

Two latent variables (ATS and PSE) have been extracted from seven observed variables (PU, PEU, PP, PC, SDT, SQ, and RD) by applying maximum likelihood factor analysis with varimax rotation.^47^ Then, internal consistency among variables has been measured with item analysis, commonly known as reliability test keeping Cronbach's alpha value as the prime consideration^48^ which is presented in *Table 3*.

**Table 3.** Results of Factor Analysis and Reliability Test

VARIABLE	FACTOR1 (ATS) LOADING	FACTOR2 (PSE) LOADING	CRONBACH'S ALPHA
PU	0.646	0.241	0.7803
PEU	0.737	0.122	
PP	0.697	0.121	
PC	0.647	0.249	
SDT	0.181	0.745	0.7997
SQ	0.203	0.797	
RD	0.173	0.668	

ATS, attitude toward the system; PSE, perceived system effectiveness.

The higher positive loadings for PU, PEU, PP, and PC indicate their strong influence on ATS. Similarly, PSE can be explained well with SDT, SQ, and RD. In both cases, the Cronbach's alpha value is higher than the standard threshold of 0.70, which indicates that the constructs of latent variables are internally consistent enough.^48^

### Correlation Among Independent Variables

A Pearson correlation matrix was prepared to test whether any multicollinearity exists among independent variables before moving them into the final model and shown in *Table 4*.

**Table 4.** Correlation Matrix of Independent Variables

	AGE	GENDER	EDU	MEX	ATS	PSE	CELLPH	ADV	SR
Age	1.00								
Gender	0.33	1.00							
Edu	−0.31	0.02	1.00						
MEx	0.21	0.16	0.32	1.00					
ATS	0.22	0.01	−0.11	−0.06	1.00				
PSE	0.15	0.14	−0.06	−0.04	0.11	1.00			
CellPh	−0.24	0.05	0.17	0.09	−0.01	0.03	1.00		
Adv	0.14	0.05	−0.03	−0.03	0.16	0.20	−0.03	1.00	
SR	0.09	0.12	−0.01	0.01	0.25	0.14	−0.08	−0.02	1.00

Adv, advertisements; CellPh, cellphone ownership; Edu, education; MEx, monthly family expenditure; SR, social reference.

The matrix shows no multicollinearity exists among independent variables since all the correlation coefficients are <0.40, which was referred as a threshold value by many researchers.^49^

### Results of Hypothesis Testing

A logistic regression modeling is used to test the hypothesis. A significance level of 0.05 is considered for this model. Decisions regarding hypothesis testing have been taken by comparing the variables' *p*-value with models' significance level. Regression coefficient indicates the nature of the relationship between independent and dependent variable, while odds ratio (OR) explains the effect of independent variables on the dependent variable. The results of hypothesis testing are shown in *Table 5*.

**Table 5.** Results of Hypothesis Testing Through Logistic Regression

H.	VARIABLE	COEF.	OR	95% CI	*p*	RESULT
1.	Age	0.062	1.0641	1.0212–1.1088	0.002	Supported
2.	Gender	—	1	—	0.033	Supported
	Female (reference)	1.003	2.7271	1.0658–6.9776		
	Male					
3.	Education				0.032	Supported
	None (reference)	—	1	—		
	Primary	2.028	7.5995	1.5170–38.0706		
	Secondary	0.925	2.5229	0.5501–11.5717		
	College & higher	0.638	1.8926	0.3588–9.9814		
4.	MEx				0.247	Not supported
	<6,000 (reference)	—	1	—		
	6001–10000	−0.330	0.7191	0.1991–2.5968		
	10,001–15,000	0.434	1.5436	0.4049–5.8842		
	15,001–20,000	−0.409	0.6644	0.1160–3.8064		
	20,000>	−1.50	0.2229	0.0211–2.3516		
5.	ATS	1.518	4.5609	2.7149–7.6620	0.000	Supported
6.	PSE	0.744	2.1046	1.3907–3.1849	0.000	Supported
7.	CellPh	1.366	3.9181	1.2850–11.9473	0.014	Supported
8.	Adv	1.937	6.9394	2.7725–17.3688	0.000	Supported
9.	SR	2.275	9.7297	4.1551–22.7834	0.000	Supported

CI, confidence interval; Coef., regression coefficient; OR, odds ratio.

The finding says that among demographic factors, age, gender, and education have significant impact on rural end-users' acceptance of e-health, while we did not find any significant influence of monthly family expenditure of the respondents on their e-health acceptance behavior. People having a positive attitude toward e-health are 4.56 times more likely to use it than those having a negative attitude. People who believe the system is effective are 2.10 times more likely to use e-health than those who do not believe. Those who have access to cellphone are 3.92 times more likely to use e-health than those who do not have. People who have exposed to Advs or promotional campaigns are 6.94 times more likely to use e-health than those who do not have. People who have SR are 9.73 times more likely to use e-health than those who do not have any reference.

### Model Summary and Goodness-of-Fit

Our model has a deviance *R^2^* of 54.70, which means that the model explains 54.70% of the deviance in the response variable. For binary logistic regression, the “Hosmer-Lemeshow” test is a more trustworthy indicator of how well the model fits the data.^50^ In this model, the goodness-of-fit score is 0.539, which is greater than the significance level of 0.05, which indicates that there is not enough evidence to conclude that the model does not fit the data.

## Discussion and Further Research Directions

This study applied an extension of TAM to determine the rural end users' acceptance behavior toward e-health in Bangladesh. We provide empirical evidence for the hypotheses in our study. Most of our findings are consistent with previous studies that applied TAM in remote healthcare systems, e-health and m-health in specific. Hoque and Bao^15^ and Davis^19^ found that PU and PEU have significant impact in forming users' attitude (ATS) toward any given system. Venkatesh et al.^20,22^ explored significant influence of output quality (SQ), RD, and SR on technology acceptance. Kim^33^ and Kayser et al.^39^ found PSE has a positive impact on system adaptability.

In our study, we found that SR has the strongest positive impact on e-health acceptance with an OR of 9.7 followed by Adv (OR = 6.9), attitude (OR = 4.5); cellphone use (OR = 3.9), and PSE (OR = 2.1). Among demographic factors, age, gender, and education were found as significant influencers, however, we did not find any significant impact of respondents' monthly family income on their acceptance of e-health. Males were found more likely to accept e-health since females in rural Bangladesh are less mobile due to social and cultural norms in a male-dominated society.^51^ In terms of education, illiterate rural people were mostly found unaware of e-health while people with an at least primary level of education have shown more interest in e-health. On the contrary, higher educated rural people considered it as a temporary alternative to the mainstream healthcare services, thus remained less interested.

The study has a few limitations. We conducted this study on a rural population of a particular geography which is Bheramara, Kushtia, a northwestern district of Bangladesh. Thus, the results may raise concerns about the generalization of the findings. Further research, therefore, can be carried out covering broader geography. Although we have extended the original TAM by adding PSE and Adv, a few additional variables could be added such as users' trust and technology anxiety to gain more comprehensive insights of e-health acceptance by rural end-users. We also believe, further longitudinal studies can be performed to observe the changes in relational pattern and strength of input variables with e-health acceptance.

## Conclusions

E-health is relatively a new phenomenon to the rural communities in developing countries. The overall success of this initiative, therefore, does not simply depend on its IT design and implementation rather large-scale users' acceptance as well.^12,18^ This study attempts to explain the factors that influence rural end-users' acceptance of e-health and found SR as the most significantly influential factor followed by Adv, users' ATS, access to a cellphone and PSE.

Thus, e-health service providers who are intended to offer their services to rural areas in developing countries should focus more on generating SRs or positive word-of-mouth. They also should conduct Adv to create mass awareness and to inform the positive features and potential benefits of e-health. To create a positive attitude toward e-health easy-to-use system, shorter SDT, and affordable price should be taken care of.

The findings of this study provide an applied guideline to the successful adoption of e-health among rural communities in developing countries. This also creates an opportunity for e-health technology developers and service providers to have a better understanding of their end users, which in turn will empower them to address the challenges in regards to the design and implementation of successful e-health initiatives.
